# A Novel Tool for the Assessment of Pain: Validation in Low Back Pain

**DOI:** 10.1371/journal.pmed.1000047

**Published:** 2009-04-07

**Authors:** Joachim Scholz, Richard J. Mannion, Daniela E. Hord, Robert S. Griffin, Bhupendra Rawal, Hui Zheng, Daniel Scoffings, Amanda Phillips, Jianli Guo, Rodney J. C. Laing, Salahadin Abdi, Isabelle Decosterd, Clifford J. Woolf

**Affiliations:** 1 Department of Anesthesia and Critical Care, Massachusetts General Hospital, Boston, Massachusetts, United States of America; 2 Academic Neurosurgery Unit, Addenbrooke's Hospital, Cambridge, United Kingdom; 3 Massachusetts General Hospital Biostatistics Center, Boston, Massachusetts, United States of America; 4 Department of Radiology, Addenbrooke's Hospital, Cambridge, United Kingdom; 5 Department of Physiotherapy Services, Addenbrooke's Hospital, Cambridge, United Kingdom; 6 Department of Anesthesiology, Perioperative Medicine, and Pain Management, University of Miami School of Medicine, Miami, Florida, United States of America; 7 Department of Anesthesiology, University Hospital Center, and Department of Cell Biology and Morphology, University of Lausanne, Lausanne, Switzerland; Imperial College London, United Kingdom

## Abstract

**Background:**

Adequate pain assessment is critical for evaluating the efficacy of analgesic treatment in clinical practice and during the development of new therapies. Yet the currently used scores of global pain intensity fail to reflect the diversity of pain manifestations and the complexity of underlying biological mechanisms. We have developed a tool for a standardized assessment of pain-related symptoms and signs that differentiates pain phenotypes independent of etiology.

**Methods and Findings:**

Using a structured interview (16 questions) and a standardized bedside examination (23 tests), we prospectively assessed symptoms and signs in 130 patients with peripheral neuropathic pain caused by diabetic polyneuropathy, postherpetic neuralgia, or radicular low back pain (LBP), and in 57 patients with non-neuropathic (axial) LBP. A hierarchical cluster analysis revealed distinct association patterns of symptoms and signs (pain subtypes) that characterized six subgroups of patients with neuropathic pain and two subgroups of patients with non-neuropathic pain. Using a classification tree analysis, we identified the most discriminatory assessment items for the identification of pain subtypes. We combined these six interview questions and ten physical tests in a pain assessment tool that we named Standardized Evaluation of Pain (StEP). We validated StEP for the distinction between radicular and axial LBP in an independent group of 137 patients. StEP identified patients with radicular pain with high sensitivity (92%; 95% confidence interval [CI] 83%–97%) and specificity (97%; 95% CI 89%–100%). The diagnostic accuracy of StEP exceeded that of a dedicated screening tool for neuropathic pain and spinal magnetic resonance imaging. In addition, we were able to reproduce subtypes of radicular and axial LBP, underscoring the utility of StEP for discerning distinct constellations of symptoms and signs.

**Conclusions:**

We present a novel method of identifying pain subtypes that we believe reflect underlying pain mechanisms. We demonstrate that this new approach to pain assessment helps separate radicular from axial back pain. Beyond diagnostic utility, a standardized differentiation of pain subtypes that is independent of disease etiology may offer a unique opportunity to improve targeted analgesic treatment.

## Abstract 2

Joachim Scholz and colleagues develop and validate an assessment tool that distinguishes between radicular and axial low back pain.

## Editors' Summary

**Background:**

Pain, although unpleasant, is essential for survival. Whenever the body is damaged, nerve cells detecting the injury send an electrical message via the spinal cord to the brain and, as a result, action is taken to prevent further damage. Usually pain is short-lived, but sometimes it continues for weeks, months, or years. Long-lasting (chronic) pain can be caused by an ongoing, often inflammatory condition (for example, arthritis) or by damage to the nervous system itself—experts call this “neuropathic” pain. Damage to the brain or spinal cord causes central neuropathic pain; damage to the nerves that convey information from distant parts of the body to the spinal cord causes peripheral neuropathic pain. One example of peripheral neuropathic pain is “radicular” low back pain (also called sciatica). This is pain that radiates from the back into the legs. By contrast, axial back pain (the most common type of low back pain) is confined to the lower back and is non-neuropathic.

**Why Was This Study Done?:**

Chronic pain is very common—nearly 10% of American adults have frequent back pain, for example—and there are many treatments for it, including rest, regulated exercise (physical therapy), pain-killing drugs (analgesics), and surgery. However, the best treatment for any individual depends on the exact nature of their pain, so it is important to assess their pain carefully before starting treatment. This is usually done by scoring overall pain intensity, but this assessment does not reflect the characteristics of the pain (for example, whether it occurs spontaneously or in response to external stimuli) or the complex biological processes involved in pain generation. An assessment designed to take such factors into account might improve treatment outcomes and could be useful in the development of new therapies. In this study, the researchers develop and test a new, standardized tool for the assessment of chronic pain that, by examining many symptoms and signs, aims to distinguish between pain subtypes.

**What Did the Researchers Do and Find?:**

One hundred thirty patients with several types of peripheral neuropathic pain and 57 patients with non-neuropathic (axial) low back pain completed a structured interview of 16 questions and a standardized bedside examination of 23 tests. Patients were asked, for example, to choose words that described their pain from a list provided by the researchers and to grade the intensity of particular aspects of their pain from zero (no pain) to ten (the maximum imaginable pain). Bedside tests included measurements of responses to light touch, pinprick, and vibration—chronic pain often alters responses to harmless stimuli. Using “hierarchical cluster analysis,” the researchers identified six subgroups of patients with neuropathic pain and two subgroups of patients with non-neuropathic pain based on the patterns of symptoms and signs revealed by the interviews and physical tests. They then used “classification tree analysis” to identify the six questions and ten physical tests that discriminated best between pain subtypes and combined these items into a tool for a Standardized Evaluation of Pain (StEP). Finally, the researchers asked whether StEP, which took 10–15 minutes, could identify patients with radicular back pain and discriminate them from those with axial back pain in an independent group of 137 patients with chronic low back pain. StEP, they report, accurately diagnosed these two conditions and was well accepted by the patients.

**What Do These Findings Mean?:**

These findings indicate that a standardized assessment of pain-related signs and symptoms can provide a simple, quick diagnostic procedure that distinguishes between radicular (neuropathic) and axial (non-neuropathic) low back pain. This distinction is crucial because these types of back pain are best treated in different ways. In addition, the findings suggest that it might be possible to identify additional pain subtypes using StEP. Because these subtypes may represent conditions in which different pain mechanisms are acting, classifying patients in this way might eventually enable physicians to tailor treatments for chronic pain to the specific needs of individual patients rather than, as at present, largely guessing which of the available treatments is likely to work best.

**Additional Information:**

Please access these Web sites via the online version of this summary at http://dx.doi.org/10.1371/journal.pmed.1000047.

- This study is further discussed in a *PLoS Medicine* Perspective by Giorgio Cruccu and and Andrea Truini
- The US National Institute of Neurological Disorders and Stroke provides a primer on pain in English and Spanish
- In its 2006 report on the health status of the US, the National Center for Health Statistics provides a special feature on the epidemiology of pain, including back pain
- The Pain Treatment Topics Web site is a resource, sponsored partly by associations and manufacturers, that provides information on all aspects of pain and its treatment for health care professionals and their patients
- Medline Plus provides a brief description of pain and of back pain and links to further information on both topics (in English and Spanish)
- The MedlinePlus Medical Encyclopedia also has a page on low back pain (in English and Spanish)

## Introduction

Conventional measures of chronic pain either rely on global scores to assess pain intensity and determine treatment success, or they employ surrogate markers of improvement such as gain of function and quality of life [1]. However, rating overall pain intensity or documenting functional improvement does not take into account the neurobiological complexity of pain [2]–[5]. Global assessments fail to reflect basic characteristics of the pain, for example, if pain occurs spontaneously or in response to external stimuli. Likewise, evaluations based on descriptions of the pain's sensory properties (pain qualities) [6], its unpleasantness, or other psychological dimensions [7] do not give any insight into its underlying mechanisms. We argue that inadequate pain assessment contributes to the difficulty of providing patients with targeted pain treatment, resulting in a high number of nonresponders. It also hampers the measurement of treatment efficacy and the development of new analgesic therapies.

Clinical pain is generally classified as acute or chronic and is divided into two major categories: inflammatory and neuropathic pain. Combinations of inflammatory and neuropathic pain do occur, for example, in postsurgical [3], cancer [4] and back pain [8]. Distinct mechanisms are responsible for the development and the persistence of clinical pain. Tissue damage causes the release of inflammatory mediators, which leads to sensitization of peripheral nociceptors. Enhanced afferent input from sensitized afferents and the spread of cytokines from inflamed tissue increase the excitability of neurons in the central nervous system (central sensitization). As a result, normally innocuous stimuli now produce pain (allodynia), and an area of heightened pain sensitivity (hyperalgesia) expands beyond the site of the inflammation [2], [9]. Neuropathic pain—defined as pain arising as a direct consequence of a lesion or disease affecting the somatosensory system [10]—also involves abnormal (ectopic) activity of primary sensory neurons and central sensitization. Altered gene transcription in primary sensory and spinal neurons, changes in synaptic connectivity, a shift from inhibition to enhanced facilitation of sensory transmission in the spinal cord, and a marked neuroimmune response are further elements of the complex process that ultimately leads to persistent neuropathic pain [11], [12].

A global pain intensity score obliterates differences in pain-related symptoms or signs that might indicate the relative contribution of particular mechanisms to a patient's pain. A clinically more useful pain assessment would associate particular symptoms and signs that constitute the pain phenotype of a patient with the underlying mechanisms and in this way reveal potential targets for pharmacological intervention. Such an assessment would provide a specific measure of treatment success or failure. It would also allow tailoring analgesic therapy to the needs of the individual patient by selecting and combining drugs with proven efficacy against operating mechanisms. However, it is currently not possible to determine pain mechanisms in patients directly [13], [14]. Techniques such as quantitative sensory testing, nerve conduction studies and evoked potentials, functional imaging, skin biopsies, and genetic screening are research tools that provide valuable information about the neurobiology of pain. But these investigations are labor-intensive, expensive, and require a level of technical expertise that is available only at highly specialized centers; they are not suitable for routine evaluation of a patient's pain or application in large clinical trials on analgesic efficacy.

We hypothesize that pain mechanisms are reflected and therefore recognizable by the specific patterns of pain-related symptoms and clinical signs they elicit [15], [16]. For example, ectopic excitability in injured sensory nerve fibers is likely to produce intermittent episodes of spontaneous pain, whereas facilitation of synaptic transmission in the dorsal horn of the spinal cord leads to pain evoked by light touch. In the present study, we have developed a tool for the assessment of neuropathic pain that is designed to differentiate subtypes of pain based on particular constellations of symptoms and signs, and tested its utility for the distinction between radicular and axial back pain.

## Methods

We conducted the study in two parts. In Part 1, we prospectively evaluated a comprehensive range of pain-related symptoms and signs in order to develop a standardized clinical tool for the differentiation of pain subtypes. We performed this part of the study at the Massachusetts General Hospital Center for Pain Medicine in Boston, Massachusetts, United States, from March 2002 until October 2004. Part 2 of the study, in which we validated the pain assessment tool, was carried out between January 2006 and November 2007 at the Back Pain Triage Clinic and the Neurosurgical Outpatient Clinic of Addenbrooke's Hospital, Cambridge, United Kingdom. The study was conducted according to the principles expressed in the Declaration of Helsinki. The study protocols were approved by the Human Research Committee of Massachusetts General Hospital (2001-P-000872) and the National Health Service Research Ethics Committee in Cambridge (05/Q0108/477). All patients gave written informed consent.

### Part 1. Development

#### Participants

We recruited patients with painful diabetic polyneuropathy (DN), postherpetic neuralgia (PHN), or chronic low back pain (LBP) through physician referrals and advertisements. Patients with DN had clinically documented diabetes mellitus, reported distal abnormal sensations such as numbness and tingling in their feet and lower legs, and exhibited symmetric sensory deficits and absent ankle jerk reflexes upon examination. If available, records of impaired nerve conduction were used to supplement the clinical diagnosis of a neuropathy, but electrophysiological evaluation was not an inclusion criterion. Patients with PHN had persistent pain in an area previously affected by an eruption of herpes zoster; altered sensation, scarring, and changes in skin pigmentation were considered additional signs supporting the diagnosis of PHN. We divided patients with chronic LBP in two groups: with (radicular LBP) and without (axial LBP) clinical signs of nerve root involvement, including sensory or motor deficits in the leg and a diminution or loss of tendon reflexes. In taking the history of patients with LBP, we asked about the primary location of the pain and whether it radiated into the leg. We also explored whether body positions such as sitting or activities such as weight-bearing or walking elicited or enhanced the pain. If available, we considered results from spinal imaging and further investigations such as electromyography for the diagnostic decision.

We included patients who met all of the following criteria: pain duration ≥3 mo, average global pain intensity in the week prior to enrollment ≥6 on a numerical rating scale (NRS) from 0 to 10, age ≥18 y, and ability to give written informed consent. Patients with a severe medical or psychiatric illness, another painful disorder or neurological disease that might have interfered with the pain assessment, or a local infection were excluded. The patients were allowed to continue their previously prescribed analgesic treatment.

#### Assessment of symptoms and signs

We evaluated pain-related symptoms and signs and somatosensory function through a structured interview followed by a standardized physical examination. The interview consisted of 16 questions exploring 46 items; the physical examination included 23 bedside tests that provided information about 39 items (see Table S1).

In the interview, we explored the location and temporal characteristics of the pain and its dependence on external stimulation. To determine the sensory quality of the pain, we first asked the patients to characterize the pain in their own words. We then offered a choice of one or more descriptions from the following list: throbbing, pounding, pulsating, shooting, radiating, cramping, squeezing, stabbing, sharp, aching, dull, painful pins and needles, stinging, burning, or hot. We also asked the patients to report unpleasant nonpainful sensations (dysesthesia) and describe the characteristics of these sensations. The patients indicated the intensity of each specific aspect of their pain using an 11-point NRS from 0 (no pain) to 10 (maximum imaginable pain) (see Table S1).

The physical examination was designed to involve tests that would be readily available for a bedside assessment, without the need for advanced or expensive technical equipment. We looked for cutaneous changes indicative of an autonomic nervous system disorder. Two von Frey filaments (North Coast Medical, Morgan Hill, California, United States) were employed to examine the response to punctate tactile stimulation: a filament of low strength (2 g) applied a force above the detection threshold for touch on intact skin; a filament of high strength (26 g) elicited a prickly, but normally painless sensation [17], [18]. Each filament was applied four times; the result was considered positive when three stimulations produced a response. The response to light pressure was tested using the eraser end of a pencil, which was applied so as just to indent the skin for 10 s. To assess the pressure sensitivity of deep tissues, the examiner applied firm pressure using her or his thumb. A soft brush (width 1 cm) was lightly moved over the skin at 3–5 cm per second in a constant direction to assess the response to dynamic tactile stimulation. To examine the response to pinprick, we used a standard safety pin and indented the skin four times with enough pressure to elicit a painful response on normal skin without leaving a mark. We recorded decreased or excess pinprick-evoked pain, respectively, when the pain was reduced or increased compared to the response to pinprick in an adjacent or contralateral unaffected area in three out of four stimulations. The sense of vibration was tested with a standard tuning fork (128 Hz). The tuning fork was applied over the first metatarsophalangeal joint for patients with DN, over the spinous process of a vertebra belonging to the spinal segment affected by PHN, or the L5 spinous process and the first metatarsophalangeal joint for patients with LBP. We employed cylindrical brass bars (diameter 1 cm) kept at 20°C to produce a nonpainful cold stimulus and 40°C for a nonpainful warm stimulus, respectively [18], [19]; each temperature probe was applied for 10 s. Proprioception was examined by testing the sense of position and passive movement. For the passive straight-leg-raising test, we lifted the affected leg extended at the knee to a 90° angle unless elevation was limited by pain; this was followed by an elevation of the leg flexed at the knee. We considered the test result positive when pain projecting into a dermatome was reproduced by raising the affected leg a second time extended at the knee [20]. To test for temporal summation, the stronger of the two von Frey filaments was applied repetitively at a rate of 1–2 Hz for 30 s [21]. If this von Frey filament elicited pain at baseline, the weaker filament was used instead.

Investigators graded a decreased response to stimulation following standardized guidelines. For example, a reduced response to punctate tactile stimulation was considered mild if the patient failed to notice stimulation with the low-strength von Frey filament but detected the high-strength filament. If pain was provoked by a test stimulus, we asked the patient to rate the intensity of this particular pain using an NRS. All tests were carried out in the affected (painful) body area. We compared the results with the response to stimulation in an adjacent or contralateral area that was free of pain or any other sensory disorder such as numbness or dysesthesia. For diabetic patients with neuropathic symptoms and signs below the knee, we chose the thigh as reference. For patients with PHN, we examined the contralateral dermatome. For patients with LBP, we examined a reference area in the midline of the back above the painful area (usually around T12), and if the patient had leg pain, the corresponding dermatome of the opposite leg.

All investigators involved in the study were experienced clinicians. Training sessions were held before patients were recruited to ensure that each investigator was performing the assessment of pain-related symptoms and signs in the same way and that the interpretation of patient responses was consistent between investigators.

#### Statistical analysis

Groups of patients with similar responses in the interview and the physical examination were identified by hierarchical cluster analysis. Using the function DAISY of R (version 2.0.1; R Foundation for Statistical Computing, Vienna, Austria; http://www.r-project.org/), we calculated a distance matrix based on Gower's general dissimilarity coefficient [22]. Binary variables were treated as asymmetric. We applied Ward's method [23] to perform a hierarchical cluster analysis on the distance matrix. In the resulting dendrogram, we set an arbitrary separation threshold so that the resulting clusters would not include fewer than approximately 10% of the total patients.

A classification tree with either a patient's clinical diagnosis or assignment to one of the patient clusters as outcome variables was derived using the RPART function of R. For classification with the diagnosis as outcome variable, the patients were randomly partitioned into a training set (two-thirds of the patients) and a test set (the remaining third), with each diagnosis represented at the same proportion in both sets as in the overall group of patients. For classification with the patient clusters as outcome variable, all patients were used to train the tree with 10-fold cross-validation. Here, we intended to determine the minimum number of interview questions and physical tests that allowed correct assignment of the patients to the clusters. We named this shorter version of the initial assessment tool Standardized Evaluation of Pain (StEP).

### Part 2. Validation

#### Participants

**Figure 1:**
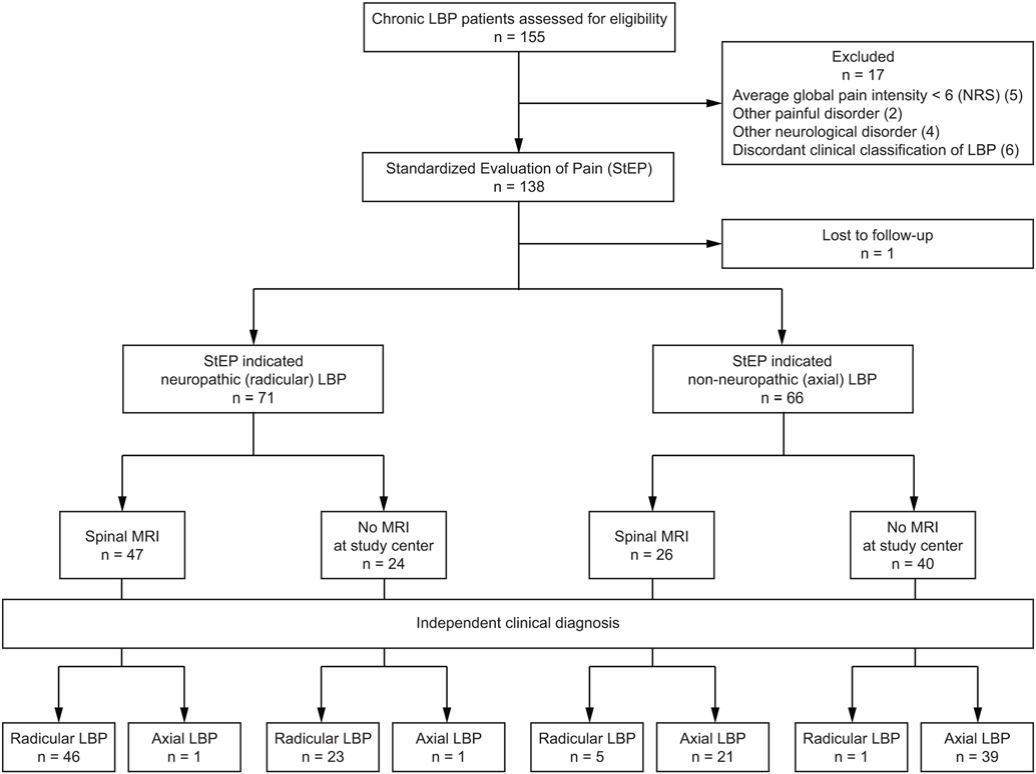
Standards for the Reporting of Diagnostic Accuracy (STARD) flowchart for the validation of StEP (Part 2 of the study).

In Part 2 of the study, we applied StEP to an independent group of patients with chronic LBP to validate the assessment tool for the distinction between radicular and axial back pain (Figure 1). The patients were recruited through physician referrals, using the same eligibility criteria as in Part 1 of the study.

#### Standardized evaluation of pain

StEP consists of a brief structured interview including six questions and ten standardized physical tests (see Text S1). To ensure a consistent application of StEP, we trained the investigators at the beginning of the validation study and provided them with detailed written instructions on how to conduct the interview, perform the physical tests, and interpret and document the findings (see Text S1).

#### Measures of diagnostic accuracy

We used the clinical diagnosis as the reference standard to determine the sensitivity and specificity of StEP and its positive and negative predictive values for the distinction between radicular and axial back pain. A patient's back pain was classified as radicular or axial by an interdisciplinary team of at least two experienced physicians—usually a rheumatologist and a neurosurgeon—and a spinal physiotherapist, who were not involved in the study. Typically, the physicians and the physiotherapist based their diagnosis on a detailed history and a comprehensive neurological and rheumatological examination that included testing for radicular sensory or motor deficits, reduced tendon reflexes, neurogenic claudication, and an evaluation of back mobility and muscle spasm. Further investigations such as electromyography, spinal magnetic resonance imaging (MRI), or computed tomography, with or without myelography, were carried out if deemed necessary. The study investigators who applied StEP were blind to the clinical classification of back pain and the results of additional investigations.

We compared StEP with the *Douleur Neuropathique en 4 Questions* (DN4) screening tool for neuropathic pain [24]. The DN4 consists of seven interview questions and three physical tests. Administration of the DN4 interview alone has been proposed for use as a self-administered questionnaire in epidemiological surveys [24]. We incorporated all ten DN4 items in the assessment in a double-blind fashion; neither the patients nor the investigators were able to differentiate between elements belonging to StEP or the DN4. Some items such as the question about the presence of burning pain were common to both assessments. Items specific to the DN4 such as the test for a decreased pricking sensation after application of a von Frey filament of 5.1 g force were grouped with similar StEP items to mask their origin. We analyzed the results of both the complete ten-item version and the seven-item interview part of the DN4 and compared them with the outcome of StEP.

#### Spinal imaging

Spinal MRI is a widely used noninvasive method to assess degenerative changes of the spine and intervertebral disk pathology leading to nerve root compression. We analyzed the MR images of those patients who were referred for the procedure as part of their diagnostic evaluation. The decision to refer a patient for spinal MRI was made independently of the study by the attending physicians. MRI of the lumbar spine was performed with a 1.5-T scanner (Signa, General Electric Healthcare, Slough, United Kingdom) and a dedicated receive-only spine coil. The imaging protocol included the following sequences: sagittal T2-weighted fast spin echo (TR 3200–3800 ms, TE 95–105 ms, 3 NEX, 512×512 matrix), sagittal T1-weighted spin echo (TR 400–820 ms, TE 9–22 ms, 3 NEX, 512×512 matrix), and axial T2-weighted fast spin echo (TR 2700–5500 ms, TE 100–118 ms, 4 NEX, 512×512 matrix). MR images of the lumbar spine at the L3/L4, L4/L5, and L5/S1 segmental levels were read by an experienced specialist in neuroradiology who was blind to the clinical diagnosis and the result of the pain assessment. Nerve root impairment was graded depending on contact between intervertebral disks and nerve roots, nerve root deviation, and compression [25]. The severity of spinal canal and lateral recess stenoses was rated from 0/3 (none) to 3/3 (marked) [26]. Changes in the signal intensity of bone marrow along the cartilaginous endplates were classified according to Modic's types 1 to 3 [27]. Degenerative disk changes were graded from I (homogenous, bright white appearance of the disk) to V (collapsed disk space) [28]. Pathological changes of the facet joints were classified on the basis of width of the facet joint space, presence of osteophytes, hypertrophy of the articular processes, subarticular bone erosions, and subchondral cysts [29].

#### Assessment of face validity

After completion of the pain assessment, we asked the patients to evaluate StEP on a standardized self-administered form. Using an NRS from 1 to 5, the patients graded the accuracy and comprehensiveness of the pain assessment, and how difficult it was for them to respond to the interview questions and comply with the physical tests. The patients also rated their willingness to repeat the pain assessment during a future visit.

#### Sample size and power

Not considering pain intensity ratings and the question about current pain, the 16 interview questions and physical tests of StEP contain 45 binary predictors. For the purpose of calculating statistical power, we constructed a composite score consisting of a linear combination of these predictors and estimated the sensitivity and specificity of StEP in distinguishing between radicular and axial back pain. We generated a receiver operating characteristic (ROC) curve for the score based on the results that we obtained in the previous part of our study. The estimated area under the ROC curve was 0.97. Assuming that the area under the curve (AUC) in the validation study would be 0.90, we calculated that a sample size of 65 patients per diagnostic group would provide 80% power to determine in a two-sided test at the 0.05 significance level whether the AUC is ≥0.80.

#### Statistical analysis

We employed the software SAS (version 9.1.3; SAS Institute, Cary, North Carolina, United States) for the statistical analysis of our validation study. Only complete patient data sets were included in the analysis.

Using the LOGISTIC procedure of SAS, we performed a logistic regression analysis to examine the relationship between StEP items and the clinical diagnosis of radicular and axial LBP. We fitted a linear logistic regression model for binary response data by the method of maximum likelihood. Based on the regression coefficients of StEP items, we created numerical scores that reflect the size of the contribution of these variables to the outcome. Potential cutoff values for the total StEP score were evaluated based on the number of correctly classified patients and the balance between sensitivity and specificity to identify patients with radicular LBP. We generated an ROC curve for the fitted model and calculated its AUC using the trapezoid rule. An ROC curve is a graphical representation of the test results with the AUC being measured in a range of 0 to 1. Values close to 1 indicate a higher power of discrimination between a positive (radicular LBP) and a negative (axial LBP) test outcome. We also constructed ROC curves for the ten-item and seven-item versions of the DN4 screening tool and for the radiological assessment of nerve root impairment by spinal MRI. To compare the AUCs of ROC curves, we generated an estimated covariance matrix based on a nonparametric approach using the theory on generalized *U*-statistics [30].

Sensitivity, specificity, and positive and negative predictive values for identifying patients with radicular back pain and the corresponding two-sided 95% confidence intervals (CIs) are provided for each diagnostic method. Areas under the ROC curves are given as mean±standard error.

## Results

We assessed 219 patients in Part 1 of the study and 155 patients in Part 2 for eligibility. Thirty-two patients in Part 1 and 11 patients in the Part 2 were excluded because the duration or average global intensity of their pain did not meet the inclusion criteria, or because they suffered from other painful disorders, or neurological or psychiatric diseases that would have compromised the evaluation of their pain. Another six patients with LBP were excluded from the validation study, because there was no unanimous decision between the attending physicians on the clinical classification of their pain as radicular or axial. One patient in the validation study was lost to follow-up because his records were incomplete. Table 1 lists the clinical characteristics of the patients included in the study.

**Table 1:** Patient characteristics.

Characteristic	Study Part 1: Development of StEP					Study Part 2: Validation	
	DN	PHN	Radicular LBP	Neuropathic Pain (Total)	Axial LBP	Radicular LBP	Axial LBP
Total number	50	23	57	130	57	75	62
Age, median (range), y	55 (38–71)	67 (45–92)	50 (20–85)	55 (20–92)	46 (19–77)	45 (20–82)	55 (24–78)
Women	27 (54)	11 (48)	28 (49)	66 (51)	35 (61)	41 (55)	35 (56)
Men	23 (46)	12 (52)	29 (51)	64 (49)	22 (39)	34 (45)	27 (44)
Pain duration, y, median (range)	4 (0.42–15)	2 (0.25–34)	4 (0.33–29)	4 (0.25–34)	5 (0.33–39)	1 (0.25–34)	5 (0.33–46)
Global pain intensity, NRS, median (range)^a^	5 (0–10)	4 (0–10)	5 (0–10)	5 (0–10)	5 (0–8)	8 (6–10)	7 (6–10)
Analgesic treatment (drugs)							
Antidepressants	4 (8)	5 (22)	7 (12)	16 (12)	4 (7)	11 (15)	13 (21)
Anticonvulsants	16 (32)	8 (35)	14 (25)	38 (29)	9 (16)	10 (13)	5 (8)
NSAIDs, acetaminophen	28 (56)	10 (43)	39 (68)	77 (59)	47 (82)	54 (72)	49 (79)
Muscle relaxants	1 (2)	0 (0)	16 (28)	17 (13)	11 (19)	1 (1)	0 (0)
Benzodiazepines	0 (0)	0 (0)	4 (7)	4 (3)	4 (7)	6 (8)	4 (6)
Opioids	12 (24)	8 (35)	28 (49)	48 (37)	26 (46)	45 (60)	41 (66)
Local anesthetics^b^	0 (0)	6 (26)	2 (4)	8 (6)	4 (7)	1 (1)	1 (2)
Glucocorticoids^b^	1 (2)	1 (4)	15 (26)	17 (13)	10 (18)	3 (4)	2 (3)
Other^c^	0 (0)	0 (0)	2 (4)	2(2)	1 (2)	1 (1)	4 (6)
Analgesic treatment (other)							
Physical therapy	7 (14)	1 (4)	32 (56)	40 (31)	27 (47)	7 (9)	12 (19)
TENS, SCS	1 (2)	0 (0)	5 (9)	6 (5)	2 (4)	3 (4)	7 (11)
Chiropractic	0 (0)	1 (4)	11 (19)	12 (9)	6 (11)	0 (0)	0 (0)
Acupuncture	7 (14)	4 (17)	6 (11)	17 (13)	6 (11)	1 (1)	0 (0)
Other^d^	1 (2)	0 (0)	4 (7)	5 (4)	1 (2)	1 (1)	3 (5)
No treatment	9 (18)	2 (9)	2 (4)	13 (10)	2 (4)	8 (11)	2 (3)

Data are presented as number (%) unless otherwise indicated.

a As reported on the day of the assessment, prior to the examination. Some patients with predominantly intermittent pain episodes were free of pain at this time (NRS = 0).

b Topical application or injection.

c Mexiletine (1), zopiclone (1), glucosamine (1), quinine (1), and botulinum toxin injections (3).

d Lumbar support (2), muscle relaxation (1), massage (1), meditation (2), yoga (2), hypnosis (1), and magnets (1).

NRS, numerical rating scale; NSAIDs, nonsteroidal anti-inflammatory drugs; SCS, spinal cord stimulation; TENS, transcutaneous electrical nerve stimulation.

### Part 1. Development of a Standardized Evaluation of Pain

#### Symptoms and signs define distinct patient subgroups

**Figure 2:**
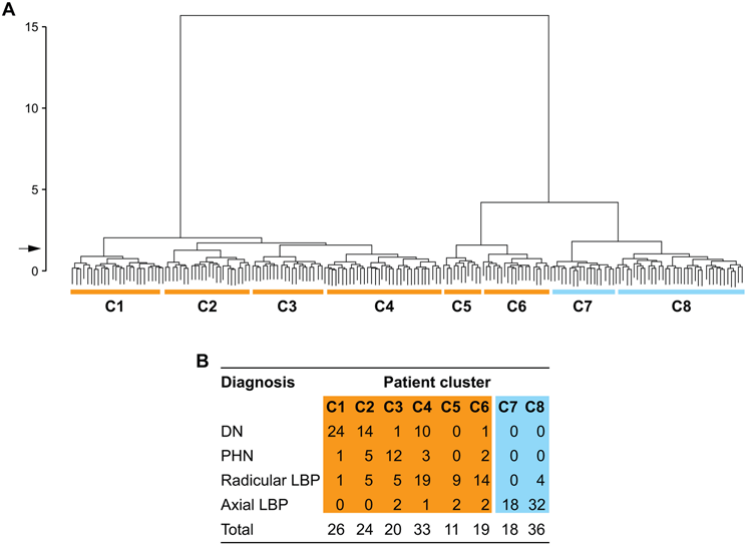
Hierarchical cluster analysis of patient subgroups defined by constellations of pain-related symptoms and signs. (A) Individual patients are symbolized by short vertical lines at the bottom of the dendrogram. Horizontal lines indicate similarities between the patients' pain, whereas upper vertical lines represent differences between pain-related signs or symptoms. At the indicated separation threshold (arrow), we identified eight subgroups of patients (clusters C1 to C8) with distinct constellations of pain-related symptoms and signs (pain subtypes). (B) Patients with DN, PHN, and radicular LBP were distributed across the clusters C1 to C6, whereas patients with axial LBP almost exclusively formed the clusters C7 and C8.

**Figure 3:**
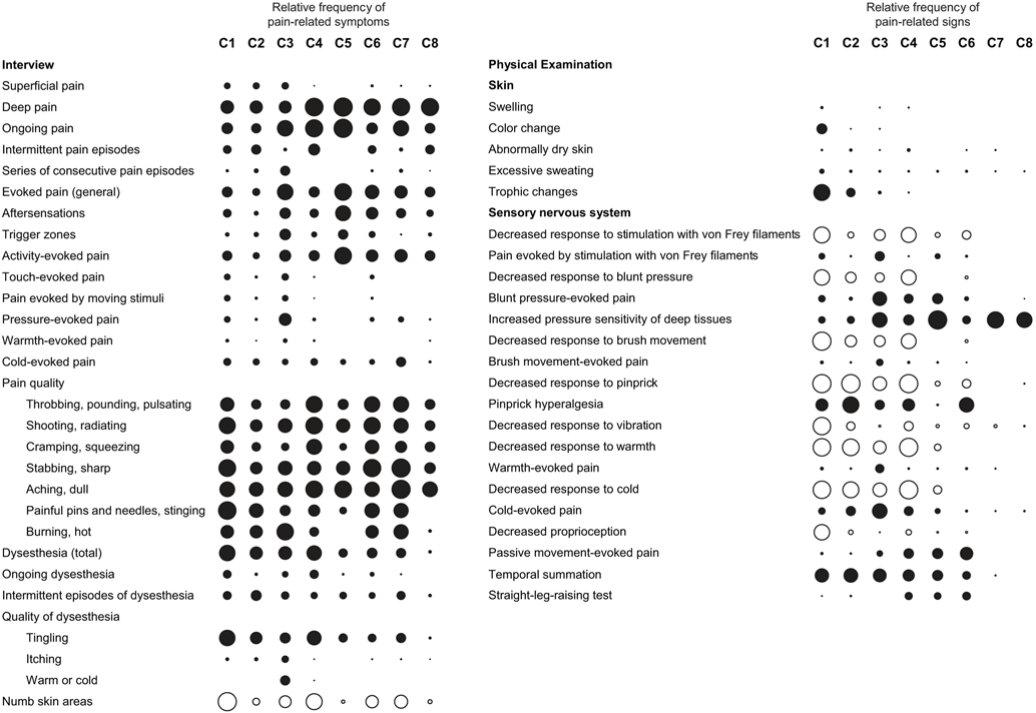
Association patterns of pain-related symptoms and signs. Circles indicate the presence of symptoms and signs, with empty circles denoting a sensory deficit. The diameter of the circles reflects the relative frequency of each symptom or sign in a patient cluster independent of the intensity of pain associated with the symptom or sign, or the severity of sensory loss. Closely related items are grouped, for example the responses to stimulation with the von Frey filaments of 2.0-g and 26.0-g strength.

We used a hierarchical cluster analysis to examine associations between pain-related symptoms and signs in the 187 patients that were included in Part 1 of our study and identified eight subgroups of patients (patient clusters) with distinct constellations of symptoms and signs (pain subtypes) (Figure 2A). The clusters C1 through C6 included the vast majority of patients with neuropathic pain, whereas patients with non-neuropathic (axial) LBP formed the clusters C7 and C8 (Figure 2B), indicating a clear difference between association patterns of symptoms and signs in patients with neuropathic and non-neuropathic pain. However, some symptoms and signs were common among patients with axial LBP and patients with radicular LBP, particularly those 23 patients with radicular LBP in clusters C5 and C6 (Figure 2). These patients exhibited, for example, a combination of deep pain, pain evoked by activity, and, in the physical examination, increased pressure sensitivity of paraspinal deep tissues that was also seen in patients with axial LBP. Patients with radicular LBP in C5 and C6 differed from those in C4 mainly because they had fewer sensory deficits. Figure 3 shows the symptoms and signs that characterized the different patient clusters.

Patients with DN, PHN, and radicular LBP were distributed across clusters C1 through C6, demonstrating that the symptoms and signs of neuropathic pain that are produced by these diseases overlap considerably (Figure 2B). Only the pain subtype represented in cluster C1 can be considered disease-specific. Twenty-four of the 26 patients in this cluster had DN (Figure 2B). Patients in this cluster reported predominantly deep pain, tingling dysesthesia, and numb skin areas. Their ability to discriminate tactile and thermal stimuli was reduced in all sensory tests. The physical examination further revealed the presence of pinprick hyperalgesia, abnormal temporal summation of repetitive stimuli, and trophic changes (Figure 3). On the other hand, we found an equal number of patients with DN in clusters C2 (14 patients) and C4 (ten patients; Figure 2B), indicating that diagnosis of a disease does not predict a particular pain subtype defined by symptoms and signs.

**Figure 4:**
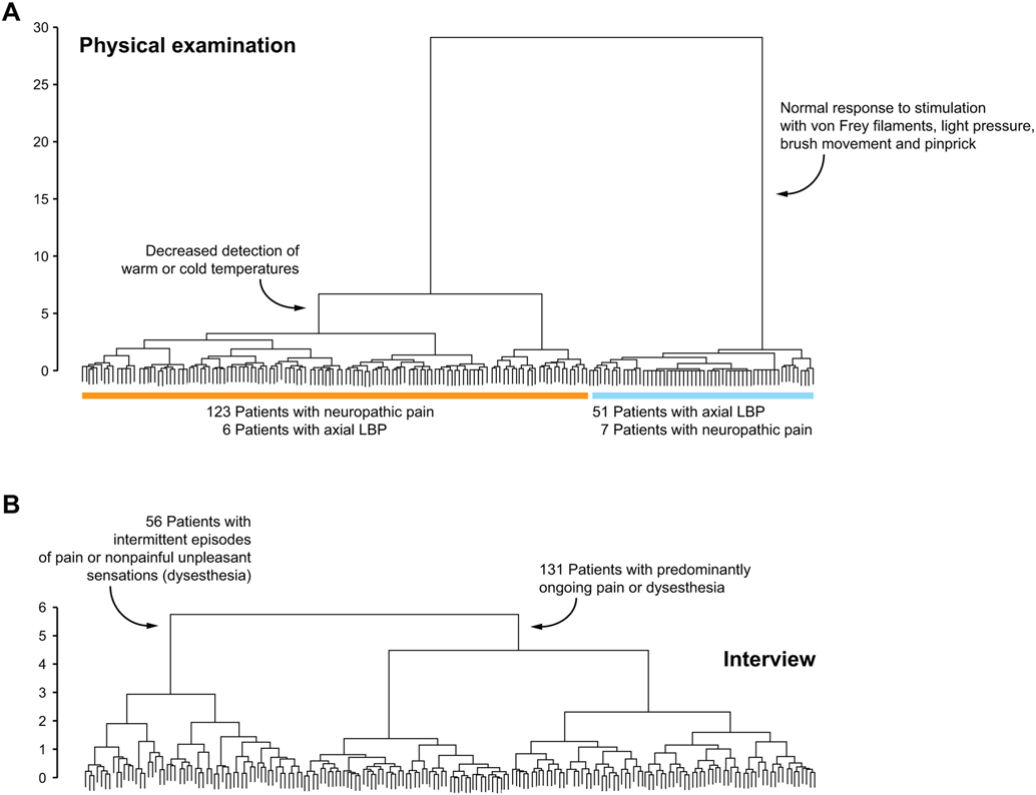
Physical examination, rather than symptom exploration, is crucial for the differentiation between patient subgroups. (A) A hierarchical cluster analysis based solely on the results of physical tests. (B) The same analysis including only pain-related symptoms reported in the interview.

The physical examination was essential for the distinction of pain subtypes. A cluster analysis based only on physical test results separated clearly between patients with neuropathic and non-neuropathic pain (Figure 4A). The results of the physical examination defined a large cluster of 129 patients, which included only six patients with axial LBP. This “neuropathic cluster” further split into two subgroups, 104 patients with decreased detection of warm or cold temperature and 25 patients with normal responses to warm and cold stimulation (Figure 4A). The “non-neuropathic cluster” of 58 patients included 51 patients with axial LBP; six patients in this cluster had radicular LBP and one patient DN. Patients in the “non-neuropathic cluster” showed normal responses to stimulation with von Frey filaments, light pressure, brush movement, and pinprick but exhibited increased sensitivity to firm pressure.

In contrast, differentiation of patient subgroups based only on symptoms described in the interview was weak and did not discriminate between patients with neuropathic and non-neuropathic pain (Figure 4B). The most prominent split here separated 131 patients with predominantly ongoing pain or dysesthesia from a group comprising 56 patients with intermittent episodes of pain or dysesthesia (Figure 4B). Subgroups within these major clusters differed by descriptions of deep versus superficial pain, the sensory quality of the pain, or numb skin areas. In the large cluster of 131 patients with ongoing symptoms, 97 patients had DN, PHN, or radicular LBP, and 34 patients had axial LBP; in the group of 56 patients with intermittent episodes of pain or dysesthesia, 33 patients had neuropathic pain, and 23 patients had axial LBP. Patients with axial LBP described a deep pain of predominantly aching or dull quality, but similar pain descriptions were recorded from patients with radicular LBP or DN (Figure 3).

#### Key characteristics of pain subtypes

**Figure 5:**
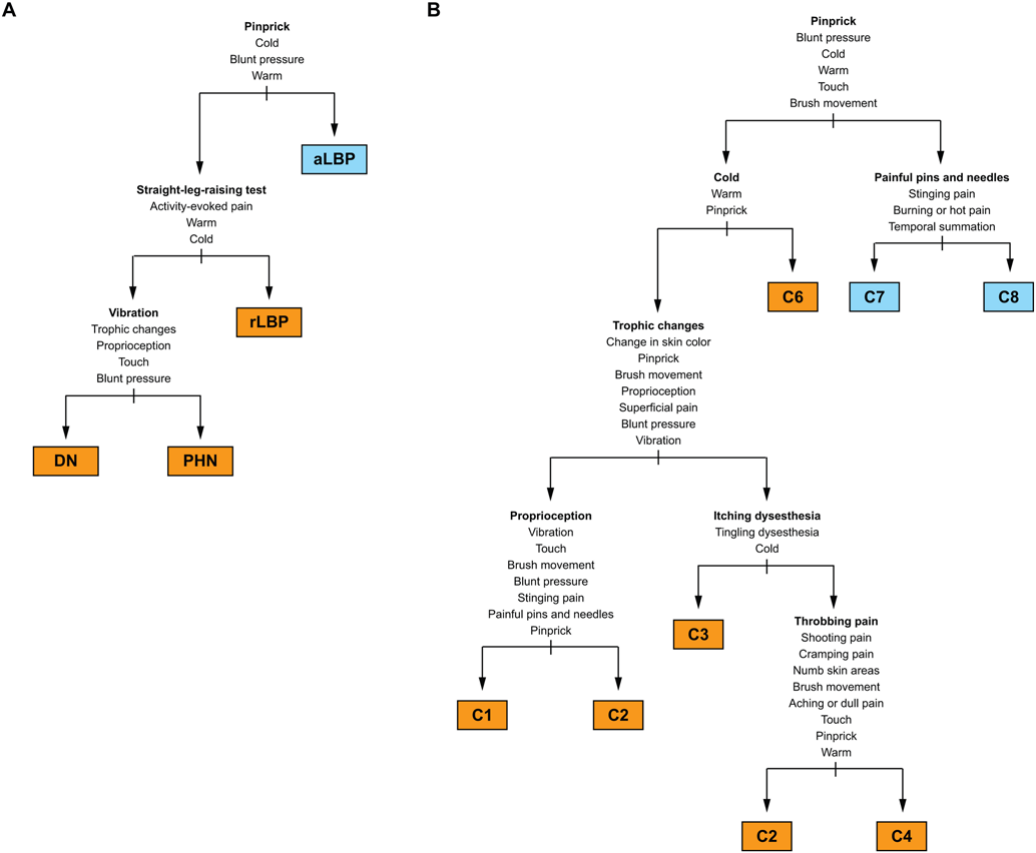
Identification of discriminatory pain assessment items. (A) Using a classification tree analysis, we determined symptoms and signs that characterized the pain in patients with DN, PHN, radicular LBP, and axial LBP. We identified an abnormal response to pinprick (either decreased response or hyperalgesia) as the best indicator of neuropathic pain. Abnormal responses to cold or warm stimuli and to blunt pressure further supported the distinction between neuropathic pain and non-neuropathic (axial) LBP. Among patients with neuropathic pain, a positive straight-leg-raising sign was closely associated with radicular LBP, and a deficit in the detection of vibration was the best marker of DN. aLBP, axial low back pain; rLBP, radicular low back pain. (B) In a separate analysis, we identified those pain assessment items that contributed to the differentiation of pain subtypes. Responses to physical tests dominated among the key characteristics of pain subtypes responsible for the allocation of patients into clusters C1 to C8. Pain assessment items in (A) and (B) are listed according to their contribution to the differentiation of painful conditions and pain subtypes, respectively. The most discriminatory items are shown on top and in bold font.

Pinprick was the most sensitive (95%; 95% CI 89%–97%) and most specific (93%; 95% CI 83%–98%) single test to distinguish between neuropathic and non-neuropathic pain. The response to pinprick was decreased or hyperalgesic in 123 of 130 patients with DN, PHN, or radicular LBP, as opposed to four out of 57 patients with axial LBP. The pinprick test must evaluate a decrease in the detection threshold; pinprick hyperalgesia alone is not a specific indicator of neuropathic pain [31]–[33]. Among patients clinically diagnosed with neuropathic pain, a positive straight-leg-raising test indicated radicular LBP with high specificity (100%). A decreased response to vibration differentiated between painful DN and PHN with a specificity of 98% and a sensitivity of 82%. These three parameters (response to pinprick, straight-leg-raising test, and response to vibration) combined had an empirical positive predictive value of 93% (95% CI 68%–99%) for painful DN, 40% (95% CI 19%–63%) for PHN, 100% (95% CI 59%–100%) for radicular LBP, and 85% (95% CI 63%–96%) for axial LBP (Figure 5A). The corresponding negative predictive values were 93% (95% CI 82%–98%), 100% (95% CI 91%–100%), 78% (95% CI 65%–88%), and 97% (95% CI 87%–99%).

Elements of the physical examination were also dominant among those variables that were identified in a classification tree analysis as key determinants of the assignment of patients into clusters: response to pinprick and cold temperature, presence of trophic skin changes, and performance in the proprioceptive tests (Figure 5B). Pain quality and the quality of dysesthesia were important for the distinction between the clusters C2, C3, and C4, and for the differentiation between the clusters C7 and C8, which comprised most of the patients with axial LBP (Figure 5B). Based on the responses to these six most discriminatory elements of the assessment alone, the probability of correct assignment of patients into clusters was 73% (95% CI 66%–79%), missing only the smallest cluster, C5, which consisted of nine patients with radicular LBP and two patients with axial LBP (Figure 2B).

Of 112 patients who described numb skin areas in the interview, 89 had a decreased response to at least one tactile or thermal test stimulus in the physical examination; 73 patients had decreased responses in both tactile and thermal tests. However, the physical examination revealed sensory deficits with higher sensitivity (in 130 patients) than the interview. In some patients skin patches with sensory loss were adjacent to areas of stimulus-evoked pain, a mixture of negative and positive signs that is a well-known feature of neuropathic pain [21], [34].

#### Standardized evaluation of pain

Based on the symptoms and signs that differentiated between the eight patient subgroups, we created a short form of the initial pain assessment tool that we named Standardized Evaluation of Pain (StEP). StEP comprises six interview questions and ten physical tests (see Text S1) that captured the key characteristics of the neuropathic pain subtypes and those features that distinguished between neuropathic and non-neuropathic pain in our patients. The application of StEP required 10–15 min, as opposed to the comprehensive assessment that included 16 interview questions and 23 tests and lasted 60–90 min.

### Part 2. Validation

Our findings indicated two possible applications for StEP, a differentiation of pain subtypes and the dichotomous distinction between neuropathic and non-neuropathic pain. Both applications are clinically valuable, yet reference standards for pain subtypes do not exist, so we decided to validate StEP for the separation between LBP with (radicular) and without (axial) involvement of the nervous system. This distinction, which has immediate consequences for therapeutic decisions [35], [36], can be challenging and often necessitates costly additional investigations. The reference standard for the validation was an independent clinical diagnosis of radicular or axial LBP achieved by an interdisciplinary team of at least two attending physicians and a spinal physiotherapist (Figure 1). Their diagnosis was typically founded on a comprehensive interview and physical examination of the patient, along with the results of additional investigations including spinal imaging.

#### Distinction between radicular and axial back pain

**Figure 6:**
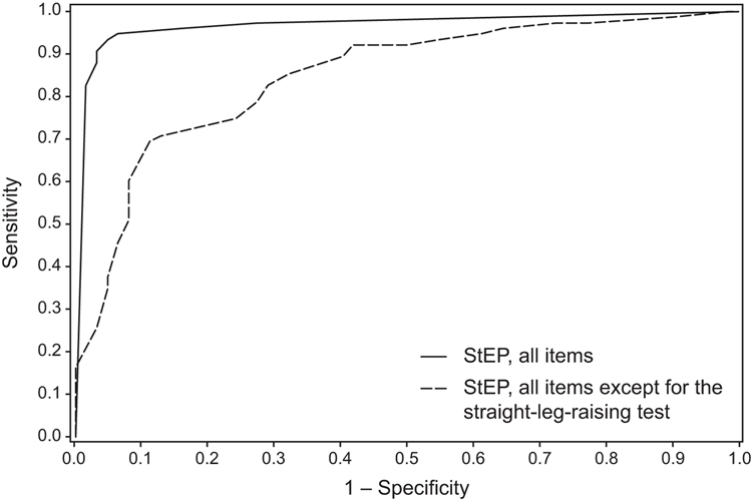
ROC curves for the distinction between radicular and axial LBP based on StEP.

We used a logistic regression analysis to determine the size of the contribution of interview questions and physical tests included in StEP to the separation between radicular and axial LBP. The results confirmed our initial observation that physical tests have more discriminatory power than interview items. A positive straight-leg-raising test and abnormal responses to cold stimulation and pinprick were key indicators of radicular LBP (Table 2). Decreased response to cold stimulation or pinprick was more important for the diagnosis of radicular LBP than cold allodynia or pinprick hyperalgesia, respectively. For example, 56 of the 75 patients with radicular LBP showed a reduced response to pinprick, compared to only 11 of the 62 patients with axial LBP, whereas 21 patients in either diagnostic group reported pinprick hyperalgesia. A burning pain quality and dynamic tactile allodynia did not constitute characteristic features of radicular LBP (Table 2), unlike peripheral neuropathic pain in other conditions [32], [37]. Based on the regression coefficients of StEP variables, we implemented a scoring system that indicates in an individual patient whether LBP is more likely to be radicular than axial (see Text S2). A cutoff value of 4 for the total score yielded 92% sensitivity (95% CI 83%–97%) and 97% specificity (95% CI 89%–100%), correctly identified 129 clinically diagnosed patients (94%), and had high positive and negative predictive values for the diagnosis of radicular LBP (Table 3). An ROC curve based on the sensitivity and specificity of StEP using this scoring system had an AUC of 0.98±0.01 (Figure 6). When the straight-leg-raising test was excluded from the analysis, the diagnostic accuracy of StEP was still high, as indicated by an area under the ROC curve of 0.85±0.03 (Figure 6).

**Table 2:** StEP scores for the distinction between radicular and axial LBP.

StEP Variable	Score
Radicular pain in the straight-leg-raising test	7
Abnormal response to cold temperature (decrease or allodynia)	3
Abnormal response to pinprick (decrease or hyperalgesia)	2
Abnormal response to blunt pressure (decrease or evoked pain)	1
Decreased response to vibration	1
Dysesthesia (any)	1
Temporal summation	−1
Burning or cold quality of the pain	−1
Abnormal response to brush movement (decrease or allodynia)	−2
Ongoing pain	−2
Skin changes (any)	−3

Scores reflect the regression coefficients of grouped StEP items; for example, a score of 2 was given when the response to pinprick was decreased or when pinprick evoked a hyperalgesic response. StEP items with a regression coefficient of 0 (zero) are not listed. A higher score is indicative of radicular LBP (see Table 3).

**Table 3:** Accuracy of StEP in identifying patients with radicular LBP at different cutoff values of the total score.

StEP Cutoff Score	Sensitivity	Specificity	Correctly Diagnosed Patients	Positive Predictive Value	Negative Predictive Value
6	81 (71–89)	100 (94–100)	123 (90)	100 (94–100)	82 (71–90)
5	88 (78–94)	97 (89–100)	126 (92)	97 (90–100)	87 (77–94)
4	92 (83–97)	97 (89–100)	129 (94)	97 (90–100)	91 (81–97)
3	93 (85–98)	94 (84–98)	128 (93)	95 (87–99)	92 (82–97)
2	96 (89–99)	82 (70–91)	123 (90)	87 (78–93)	94 (85–99)
1	97 (91–100)	69 (56–80)	116 (85)	79 (70–87)	96 (85–99)
0	99 (93–100)	52 (39–65)	106 (77)	71 (61–80)	97 (84–100)

Correctly diagnosed patients are given as number (%). All other values represent % (95% CI).

We compared StEP with a screening tool for neuropathic pain, the DN4 [24], which consists of seven interview questions and three physical tests. The physical tests assess whether sensibility to a brush touching the skin and the pricking sensation elicited by a von Frey filament are decreased, and whether movement of a brush over the skin produces a painful response. A short version of the DN4 comprises only the seven interview items [24]. The sensitivity of the ten-item version of the DN4 in our study was 61% (95% CI 49%–72%) and its specificity 73% (95% CI 60%–83%). Ninety-one patients (66%) were accurately identified as having radicular or axial LBP (Table 4). The seven interview items of the DN4 provided an accurate diagnosis in 86 patients (63%); sensitivity and specificity of the seven-item version of the DN4 were 68% (95% CI 56%–78%) and 56% (95% CI 43%–69) (Table 4). The areas under the ROC curves±standard errors for the ten-item and the seven-item versions of the DN4 were 0.71±0.04 and 0.67±0.05, respectively (see Figure S1), significantly lower than the area under the ROC curve for StEP independent of whether the straight-leg-raising test was included in the analysis of StEP's diagnostic accuracy (*p*<0.001 for either version of the DN4) or not (*p*<0.01 for the ten-item version of the DN4, and *p*<0.001 for the seven-item version).

**Table 4:** Diagnostic accuracy of StEP for the identification of radicular LBP compared to the DN4 screening tool for neuropathic pain and spinal MRI.

Measure of Accuracy	StEP (All Patients)	DN4, Ten Items	DN4, Seven Items	StEP (Patients with MRI)	Spinal MRI^a^
AUC, mean±standard error	0.98±0.01	0.71±0.04^b^	0.67±0.05^b^	0.97±0.02	0.69±0.07^b^
Sensitivity	92 (83–97)	61 (49–72)	68 (56–78)	90 (79–97)	86 (74–94)
Specificity	97 (89–100)	73 (60–83)	56 (43–69)	95 (77–100)	41 (21–64)
Correctly diagnosed patients, number (%)	129 (94)	91 (66)	86 (63)	67 (92)^c^	53 (73)^c^
Positive predictive value	97 (90–100)	73 (60–83)	65 (54–76)	98 (89–100)	77 (64–87)
Negative predictive value	91 (81–97)	61 (49–72)	59 (46–72)	81 (61–93)	56 (30–80)

Values represent % (95% CI) unless otherwise noted.

a Using deviation of a nerve root caused by disk herniation and moderate stenosis (≥2/3) of the spinal canal or a lateral recess as cutoff values.

b *p*<0.001, when compared to the area under the ROC curve for StEP.

c The spinal MR images of 73 patients were analyzed.

A large number of patients with radicular and axial LBP were included in Part 1 of the study. For an independent evaluation of the logistic regression model that we used to derive the scoring system for StEP, we applied the scores retrospectively to equivalent items of the initial pain assessment tool and determined how back pain would have been classified in these patients. We found that 89% of the patients would have been diagnosed correctly as having radicular or axial LBP, and that the sensitivity and specificity in discriminating between the two groups of patients would have been 79% (95% CI 63%–90%) and 98% (95% CI 89%–100%), respectively. These numbers underline the diagnostic utility of the scoring system. Its reduced sensitivity when applied retrospectively is likely explained by differences between StEP and the assessment tool that we employed to evaluate pain-related symptoms and signs in Part 1 of our study. This initial tool contained more interview questions and physical tests, and there were also minor differences in the wording of questions and test instructions.

#### Comparison with spinal MRI

Fifty-one patients with radicular LBP and 22 patients with axial LBP were examined by spinal MRI. Table S2 lists the radiological findings for the two patient groups. We considered nerve root impairment by a herniated intervertebral disk [25] and stenosis of either the spinal canal or a lateral recess indicators of radicular pain [26]. MRI of the lumbar spine had 96% sensitivity (95% CI 87%–100%) but only 18% specificity (95% CI 5%–40%) in identifying patients with radicular LBP when any contact of disk material with a nerve root and a spinal canal or lateral recess stenosis of ≥1/3 were regarded indicators of nerve root involvement. The specificity increased to 41% (95% CI 21%–64%) when higher cutoff scores (deviation of a nerve root and ≥2/3 stenosis of the spinal canal or a lateral recess) were applied (see Figure S2). With these stricter criteria, the sensitivity of the MRI was still high with 86% (95% CI 74%–94%), but the corresponding ROC curve had an AUC of only 0.69±0.06 (Table 4). In the subset of patients who were examined by MRI, StEP distinguished between radicular and axial LBP with a sensitivity of 90% (95% CI 79%–97%) and a specificity of 95% (95% CI 77%–100%), providing substantially higher diagnostic accuracy than MRI (Table 4).

The severity of vertebral endplate abnormalities, intervertebral disk degeneration, and facet joint arthrosis was similar in patients with radicular LBP and patients with axial LBP (see Table S2).

#### Subtypes of low back pain

**Figure 7:**
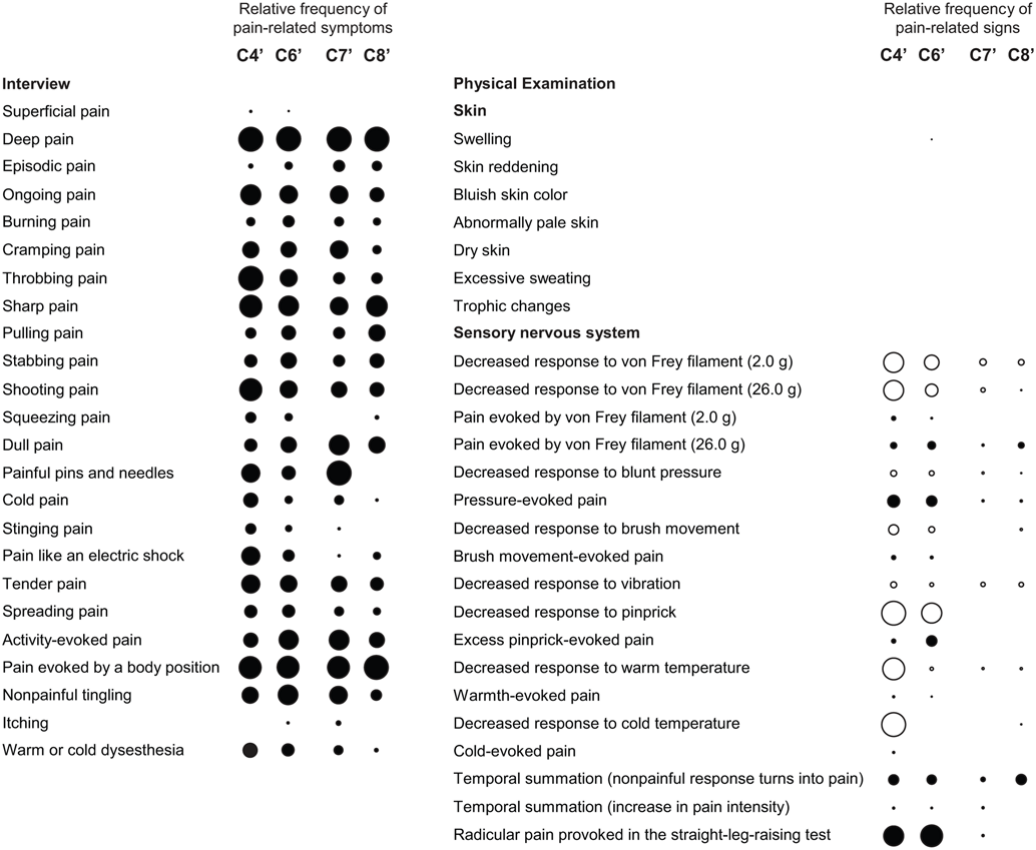
Association patterns of symptoms and signs in patients with chronic LBP in Part 2 of the study. Subgroups of patients with radicular and axial LBP were identified based on those symptoms and signs that characterized the patient clusters C4, C6, C7, and C8 in Part 1 of the study (compare Figure 5B).

Although the primary aim of the validation was to determine the sensitivity and specificity of StEP in distinguishing radicular from axial LBP, we sought to identify patients with those subtypes of LBP that we had characterized in Part 1 of our study. Based on the criteria specified in the previous classification tree analysis (Figure 5B), we found 12 patients with radicular LBP who exhibited symptoms and signs analogous to those of patients in previous cluster C4, most prominently sensory deficits in response to tactile and thermal stimuli. Symptoms and signs in another 44 patients with radicular LBP matched the pain characteristics of patients in previous cluster C6 (Figure 7). And in 11 and 21 of the patients with axial LBP we found association patterns of symptoms and signs analogous to those observed in the previous clusters C7 and C8, respectively (Figure 7). LBP in these two patient subgroups differed mainly by its sensory quality, for example the presence of “painful pins and needles.” Considering the limited discriminatory power of pain qualities, the subtypes of axial LBP that we identified in Part 1 of our study might not be as robust as those of radicular LBP. Overfitting of the classification tree to these symptoms would explain why only half of the patients with axial LBP in Part 2 of the study matched the classification criteria for clusters C7 or C8.

#### Face validity of StEP

StEP was evaluated by 134 patients. These patients regarded StEP as a suitable and appropriate tool for the assessment of their pain. StEP's comprehensiveness was rated 5 (median; lower quartile, Q_1_ = 4; upper quartile, Q_3_ = 5) on a numerical scale of 1 (many important aspects of the pain were missed) to 5 (very good representation of the pain). The ease of answering the interview questions was rated 5 (Q_1_ = 4; Q_3_ = 5) on a scale of 1 (very difficult) to 5 (very easy). The ease of compliance with the physical examination also scored as 5 (Q_1_ = 5; Q_3_ = 5). The willingness to complete the assessment again as a measure of change after treatment was 5 (Q_1_ = 5; Q_3_ = 5) on an NRS from 1 to 5, indicating high acceptance of the assessment tool. We used a two-tailed Wilcoxon rank sum test to compare how patients with radicular LBP and those with axial LBP evaluated StEP and found no significant difference in their assessment of StEP's comprehensiveness (*p* = 0.96), the ease of answering the interview questions (*p* = 0.69), or ease of compliance with the physical tests (*p* = 0.56). Patients in both groups indicated that they would be willing to complete StEP again (*p* = 0.17).

## Discussion

Chronic pain is a complex experience comprising the sensation of pain itself as well as autonomic responses, psychological reactions, and social consequences [1]. Here we explored commonalities and differences in the sensory components of peripheral neuropathic and non-neuropathic pain. Using a structured interview and a standardized physical examination, we identified and characterized subtypes of chronic pain independent of etiological disease categories. We did not attempt to measure disturbances in affect, behavior, or quality of life, for which other assessment tools are available [1]

We found that relatively few symptoms and signs can differentiate a set of distinct neuropathic pain subtypes, and that these are not defined by the condition causing the pain. Somewhat surprisingly for a sensory disorder, the physical examination was more sensitive than the exploration of symptoms for the distinction between subtypes of neuropathic pain and the separation between neuropathic and non-neuropathic pain. The quality of the pain was certainly less important than suggested by previous methods that relied exclusively on a patient interview. But the most discriminatory tests, as identified by a classification tree analysis, were generally familiar and not unexpected, such as pinprick for the detection of a sensory deficit or hyperalgesia [31], [33].

Based on their contribution to the identification of pain subtypes, we created a tool for a standardized assessment of pain that consists of six interview questions and ten physical tests, which are easily applicable in a bedside examination. We hypothesize that pain subtypes characterized by distinct patterns of these pain-related symptoms and signs indicate active biological mechanisms. Spontaneous burning pain, for example, may be driven by ectopic discharges in heat-sensitive nociceptor neurons, whereas pain evoked by brush stroke (dynamic tactile allodynia) is more likely to result from an increase in the excitability of dorsal horn neurons [9], [11]. Special investigations including the quantification of sensory fiber loss in skin biopsies [38], electrophysiological examinations of nociceptive pathways [39], and functional brain imaging [40] are critical for the elucidation of the neurobiology of pain in humans, but they are not suitable for routine clinical testing. The requirement of technical equipment, special expertise, and a substantial expenditure in time limit the use of quantitative sensory testing to evaluate somatosensory function to research studies involving small patient samples [41]. As a consequence, no major clinical trial to date has systematically examined the features of neuropathic pain and, more specifically, their relationship with treatment response or capacity to predict the response.

Patients with neuropathic pain are usually classified based on disease diagnosis. However, we did not find a unique correlation of neuropathic pain-related symptoms and signs with disease except for one pain subtype associated with a subgroup of patients with DN. Disease itself does not predict the occurrence or natural course of neuropathic pain, nor do the etiological factors and pathological changes that define a neurological disease necessarily correlate with mechanisms responsible for the manifestation of spontaneous pain, hyperalgesia, or allodynia, all common features of neuropathic pain [15], [37]. Different pain mechanisms may operate in patients with the same disease, the same pain mechanisms can be present in patients with different diseases, and the relative contribution of particular mechanisms to the pain in individual patients may change over time [5]. In addition, changes in the nervous system can become autonomous and persist long after the primary disease has disappeared, for example in postherpetic neuralgia.

We show that a standardized evaluation of pain-related symptoms and signs helps separate patients with neuropathic pain including radicular LBP from those with non-neuropathic (axial) back pain. Distinguishing between radicular and axial LBP is often difficult: nerve root involvement may manifest with a minor sensory or motor deficit, and patients with LBP originating in the facet joints or other structures of the spine may experience pain lateral to the midline, which can be confused with radicular pain. In addition, degenerative changes of the spine are likely to contribute also to back pain in patients with nerve root involvement [8], [35]. However, the differentiation between radicular and axial LBP is clinically important and has direct impact on therapeutic decisions: anticonvulsants and antidepressants are adjuvant pharmacological treatment options in neuropathic back pain [42], and patients with persistent radicular pain or neurological deficits benefit from surgical intervention [36].

We validated StEP for its ability to distinguish radicular from axial LBP and found that StEP identifies patients with radicular LBP with high diagnostic accuracy. Based on the results of our validation study, we propose a scoring system that can be implemented in the analysis of StEP to detect radicular back pain. The most discriminatory indicators for radicular pain were a positive straight-leg-raising sign, a deficit in the detection of cold, and a reduced response to pinprick. This is not too surprising: the straight-leg-raising test is routinely performed in the examination of patients with back pain [8], and demonstration of a sensory deficit in the innervation territory of a lesioned nervous structure is a diagnostic criterion of neuropathic pain [10]. Standardized application and interpretation substantially improve the diagnostic utility of both the straight-leg-raising test and the assessment of sensory function, whereas evaluation of sensory abnormalities without defined stimuli increases the variability of outcomes [14]–[16]. Radicular pain in a positive straight-leg-raising test is probably caused by traction on an impinged nerve root and may be enhanced by local edema, inflammation of the affected nerve root, or venous blood flow obstruction [20]. Differences in the procedure and the interpretation of the straight-leg-raising test are likely to account for conflicting conclusions on its diagnostic utility in clinical practice [20], [43]. Evaluations of the test further depend on the reference standard used. Some studies compared its sensitivity and specificity to a radiological assessment of nerve root impairment in spinal MRI [44]. The gold standard for the distinction between radicular and axial LBP should, however, be a conclusive clinical diagnosis that draws on several sources of information including if applicable—MRI or computed tomography, electrophysiological investigations, and surgical records [10], [37].

The DN4 screening tool for neuropathic pain was developed in a study not involving patients with radicular LBP [24]. The complete version of the DN4 contains three physical tests, for a reduced sensibility to a brush touching the skin, a decreased pricking sensation elicited by a von Frey filament, and a painful response to brush movement. However, like other screening tools for neuropathic pain [45]–[47], the DN4 relies largely on a structured exploration of the patient's history. Pain assessment tools that comprise solely interview questions or combine a questionnaire and self-administered tests [48] have advantages for use in epidemiological studies but, as our results suggest, they may lack sensitivity and specificity when applied in clinical practice. Generally these questionnaires are constructed as a short list of items that have been selected a priori based on clinical experience, with the assumption that these items will constitute useful measures for the assessment of pain. Our approach was quite different. We analyzed a comprehensive range of pain-related symptoms and signs without any presumption of their clinical importance. We found that physical tests are more useful for identifying patients with neuropathic back pain than interview questions, and that only a standardized assessment of both symptoms and signs allows a differentiation between subtypes of pain.

Further studies are needed to determine the accuracy of StEP for the distinction of neuropathic and non-neuropathic pain in conditions other than LBP. The discriminatory value of single variables will certainly vary depending on each condition. However, we believe that the interview questions and physical tests included in StEP will be sufficient, because StEP was still highly sensitive and specific in discriminating between radicular and axial LBP after excluding the straight-leg-raising sign, which has utility only for the diagnosis of radicular LBP. Future studies will also have to address important issues such as test-retest and intra- and inter-rater reliability, which we did not investigate here. Whether successful analgesic treatment modifies the presence or intensity of specific painful symptoms or signsremains to be investigated, but the reliability of StEP is likely to be affected by the response to treatment. It is therefore possible that we would have observed different constellations of symptoms and signs in untreated patients, of whom only a few were included in the present study (Table 1).

Spinal imaging is recommended for the evaluation of patients who have LBP that persists beyond 4 weeks, exhibit severe or progressive neurologic deficits, or are suspected of having a serious underlying condition such as vertebral infection or cancer [35]. MRI is the preferred technique, because it depicts intervertebral disks, nerve roots, and the spinal canal better than computed tomography and does not expose the patient to ionizing radiation. However, the specificity of MRI in the identification of nerve root involvement is reduced by the prevalence of degenerative changes of the spine in asymptomatic individuals [49]. Studies comparing clinical and radiological findings demonstrated that the degree of disk displacement in MRI correlates with outcome in the straight-leg-raising test, but not or only poorly with the severity of radicular pain or motor or sensory deficits [50]. We found that spinal MRI is a sensitive diagnostic tool for the identification of radicular LBP, but its specificity was lower than that of two clinical methods, StEP and DN4, despite the application of standardized evaluation criteria. Higher cutoff values for the radiological assessment of nerve root impairment improved the specificity of MRI to some extent, but any findings will always need to be evaluated in their clinical context [35].

We demonstrate that a standardized assessment of pain-related symptoms and signs provides a simple diagnostic procedure for the distinction between radicular and axial LBP. This distinction is crucial because back pain is a diagnostic label for a heterogeneous group of patients and it is often difficult to decide which patients will benefit from treatment strategies that target neuropathic pain. However, the potential therapeutic implications of a standardized method to identify pain subtypes go beyond the dichotomous separation between neuropathic and non-neuropathic pain. First-line medications recommended for neuropathic pain include anticonvulsants, tricyclic antidepressants, and opioids [51]–[53]; they are effective only in a proportion of the patients and reduce pain by ≥50% in only 25%–50% of the cases [53], [54]. As predictors of treatment response or failure are unknown, therapeutic decisions are largely based on empirical criteria and the presence of comorbidities [51]–[53]. Differences between underlying pain mechanisms are one possible explanation for the variability of treatment response among patients with chronic pain [55]. Classifying patients according to subtypes of pain offers the possibility of testing if treatment response correlates with the association patterns of symptoms and signs that define the subtypes [55], [56]. We hypothesize that these patterns reflect pain mechanisms and, consequently, constitute predictors of treatment efficacy.

## Supporting Information

Figure S1 ROC curves reflecting the sensitivity and specificity of the DN4 screening tool for neuropathic pain in distinguishing radicular and axial LBP.
(0.52 MB EPS)

Figure S2 Spinal MRI in patients with LBP. (A) An axial T2-weighted fast spin echo image through the vertebrae L5 and S1 shows a central disk protrusion (arrow) compressing the left S1 nerve root (arrowhead). (B) Marked stenosis (3/3) of the right lateral recess in an axial T2-weighted fast spin echo image through the facet joints of the L4 and L5 vertebrae. (C) An axial T2-weighted fast spin echo image through the L4 and L5 vertebrae indicates bilateral severe (grade 3) arthrosis of the facet joints.
(1.00 MB EPS)

Table S1 Structure of the initial pain assessment that was used to develop StEP.
(0.07 MB DOC)

Table S2 Radiological assessment of nerve root involvement and degenerative changes of spinal structures.
(0.07 MB DOC)

Text S1 Standardized evaluation of pain (StEP).
(0.10 MB PDF)

Text S2 StEP score sheet for the distinction between radicular and axial low back pain.
(0.07 MB PDF)

## Abbreviations

AUC: area under the curve
CI: confidence interval
DN: diabetic polyneuropathy
DN4: *Douleur Neuropathique en 4 Questions*
LBP: low back pain
MRI: magnetic resonance imaging
NRS: numerical rating scale
PHN: postherpetic neuralgia
ROC: receiver operating characteristic
StEP: Standardized Evaluation of Pain
