# Supplementary material for: A Novel Tool for the Assessment of Pain: Validation in Low Back Pain
Source: PLoS Med. 2009 Apr 7;6(4):e1000047. doi: 10.1371/journal.pmed.1000047 (PMC2661253; doi:10.1371/journal.pmed.1000047)
Supplement: Table S1 — Structure of the initial pain assessment that was used to develop StEP. (0.07 MB DOC) [file pmed.1000047.s003.doc]

**Table S1.** Structure of the Initial Pain Assessment that Was Used to Develop StEP

| **Interview Questions** | Response Type | **Pain intensity, degree of decrease** |
| --- | --- | --- |
| Superficial/deep pain/pain felt as if in an internal organ | MC |  |
| Ongoing pain | Y/N | NRS |
| Spontaneous worsening of ongoing pain | Y/N | NRS |
| Frequency of spontaneous worsening | MC |  |
| Intermittent pain episodes | Y/N | NRS |
| Frequency | MC |  |
| Duration | MC |  |
| Series of consecutive pain episodes | Y/N |  |
| Duration of such series | MC |  |
| Evoked pain (general) | Y/N |  |
| Aftersensations (pain persisting after the stimulation) | Y/N |  |
| Trigger zones | Y/N |  |
| Activity-evoked pain | Y/N | NRS |
| Touch-evoked pain | Y/N | NRS |
| Pain evoked by moving tactile stimuli | Y/N | NRS |
| Pressure-evoked pain | Y/N | NRS |
| Warm-evoked pain | Y/N | NRS |
| Cold-evoked pain | Y/N | NRS |
| Pain during urination or defecation | Y/N | NRS |
| Pain quality | Free and MC |  |
| Unpleasant nonpainful sensations (dysesthesia) | Y/N |  |
| Ongoing presence | Y/N |  |
| Manifesting in intermittent episodes |  |  |
| Frequency (if occurring intermittently) | Y/N |  |
| Stimulus-evoked dysesthesia | Y/N |  |
| Quality of dysesthesia | Free |  |
| Numb skin areas | Y/N |  |

Table continues on the following page.

**Table S1.** (Continued)

| **Physical Examination** | **ResponseType** | **Pain Intensity, Degree of Decrease** |
| --- | --- | --- |
| **Skin** |  |  |
| Skin lesion | MC |  |
| Swelling | Y/N |  |
| Color change | MC |  |
| Abnormally dry skin/excessive sweating | MC |  |
| Trophic changes | Y/N |  |
| **Sensory nervous system** | Y/N |  |
| Decreased response to stimulation with von Frey filaments | Y/N | Graded |
| Pain evoked by stimulation with von Frey filaments | Y/N | NRS |
| Decreased response to blunt pressure | Y/N | Graded |
| Blunt pressure-evoked pain | Y/N | NRS |
| Increased pressure sensitivity of deep tissues | Y/N | NRS |
| Decreased response to brush movement | Y/N | Graded |
| Brush movement-evoked pain | Y/N | NRS |
| Decreased response to pinprick | Y/N | Graded |
| Pinprick-hyperalgesia | Y/N | NRS |
| Decreased response to vibration | Y/N |  |
| Decreased response to warm | Y/N | Graded |
| Warm-evoked pain | Y/N | NRS |
| Decreased response to cold | Y/N | Graded |
| Cold-evoked pain | Y/N | NRS |
| Decreased proprioception (position, passive movement) | Y/N | Graded |
| Passive movement-evoked pain | Y/N | NRS |
| Straight-leg-raising test | Y/N |  |
| Temporal summation | Y/N | Graded |

Free, free response; Graded, decrease rated using standardized non-numerical grades such as mild, moderate, severe; MC, multiple choice; NRS, numerical rating scale; Y/N, yes/no or present/absent or positive/negative.
