# Supplementary material for: A Novel Tool for the Assessment of Pain: Validation in Low Back Pain
Source: PLoS Med. 2009 Apr 7;6(4):e1000047. doi: 10.1371/journal.pmed.1000047 (PMC2661253; doi:10.1371/journal.pmed.1000047)
Supplement: Table S2 — Radiological assessment of nerve root involvement and degenerative changes of spinal structures. (0.07 MB DOC) [file pmed.1000047.s004.doc]

**Table S2.** Radiological Assessment of Nerve Root Involvement and Degenerative Changes of Spinal Structures

| **Criterion** | **Degree** | **Radicular LBP (n = 51)** | | |  | **Axial LBP (n = 22)** | | |
| --- | --- | --- | --- | --- | --- | --- | --- | --- |
|  |  | **L3/L4** | **L4/L5** | **L5/S1** |  | **L3/L4** | **L4/L5** | **L5/S1** |
| Nerve root impairment by disk herniation^a^ | 0 | 36 (71) | 27 (53) | 14 (27) |  | 16 (73) | 15 (68) | 13 (59) |
|  | 1 | 3 (6) | 7 (14) | 8 (16) |  | 0 (0) | 2 (9) | 3 (14) |
|  | 2 | 0 (0) | 7 (14) | 3 (6) |  | 1 (5) | 2 (9) | 1 (5) |
|  | 3 | 0 (0) | 10 (20) | 26 (51) |  | 2 (9) | 3 (14) | 5 (23) |
| Stenosis of the spinal canal^b^ | 0/3 | 34 (67) | 33 (65) | 34 (67) |  | 15 (68) | 13 (59) | 18 (82) |
|  | 1/3 | 4 (8) | 11 (22) | 12 (24) |  | 1 (5) | 4 (18) | 1 (5) |
|  | 2/3 | 1 (2) | 7 (14) | 4 (8) |  | 1 (5) | 3 (14) | 2 (9) |
|  | 3/3 | 0 (0) | 0 (0) | 1 (2) |  | 2 (9) | 2 (9) | 1 (5) |
| Stenosis of a lateral recess^b^ | 0/3 | 35 (69) | 26 (51) | 20 (39) |  | 15 (68) | 12 (55) | 14 (64) |
|  | 1/3 | 3 (6) | 9 (18) | 4 (8) |  | 1 (5) | 5 (23) | 1 (5) |
|  | 2/3 | 0 (0) | 6 (12) | 8 (16) |  | 0 (0) | 3 (14) | 3 (14) |
|  | 3/3 | 1 (2) | 10 (20) | 19 (37) |  | 3 (14) | 2 (9) | 4 (18) |
| End plate abnormalities^c^ | 0 | 36 (71) | 44 (86) | 33 (65) |  | 17 (77) | 20 (91) | 15 (68) |
|  | 1 | 0 (0) | 0 (0) | 2 (4) |  | 0 (0) | 0 (0) | 0 (0) |
|  | 2 | 3 (6) | 6 (12) | 14 (27) |  | 2 (9) | 2 (9) | 7 (32) |
|  | 3 | 0 (0) | 0 (0) | 1 (2) |  | 0 (0) | 0 (0) | 0 (0) |
| Disk degeneration^d^ | I | 2 (4) | 1 (2) | 1 (2) |  | 1 (5) | 1 (5) | 0 (0) |
|  | II | 10 (20) | 9 (18) | 2 (4) |  | 2 (9) | 1 (5) | 3 (14) |
|  | III | 16 (31) | 10 (20) | 7 (14) |  | 8 (36) | 8 (36) | 5 (23) |
|  | IV | 8 (16) | 27 (53) | 27 (53) |  | 7 (32) | 11 (50) | 6 (27) |
|  | V | 3 (6) | 3 (6) | 13 (25) |  | 1 (5) | 1 (5) | 8 (36) |
| Facet joint arthrosis^e^ | 0 | 13 (25) | 5 (10) | 8 (16) |  | 6 (27) | 2 (9) | 5 (23) |
|  | 1 | 23 (45) | 33 (65) | 34 (67) |  | 8 (36) | 11 (50) | 15 (68) |
|  | 2 | 3 (6) | 9 (18) | 5 (10) |  | 4 (18) | 7 (32) | 1 (5) |
|  | 3 | 1 (2) | 4 (8) | 3 (6) |  | 1 (5) | 2 (9) | 1 (5) |

Data are presented as number of patients (%). Differences to 100% indicate that the respective anatomical level was not included in the MRI.

^a^ The degree of the impairment was graded 0 (none) to 3 depending on contact of the nerve root with the intervertebral disk, nerve root deviation and compression [1].

^b^ Spinal canal and lateral recess stenoses were rated from 0 (none) to 3/3 (severe) [2].

^c^ Changes in the signal intensity of vertebral bone marrow along the cartilaginous endplates were classified according to Modic’s types 1 to 3 [3].

^d^ Disk degeneration was graded I (homogenous, bright white appearance of the disk) to V (collapsed disk space) [4].

^e^ Facet joint arthrosis was classified based on the width of the facet joint space, the presence of osteophytes, hypertrophy of the articular processes, subarticular bone erosions and subchondral cysts [5].

References cited in the footnote of Table S2 are listed on the following page.

**References**

1. Pfirrmann CW, Dora C, Schmid MR, Zanetti M, Hodler J, et al. (2004) MR image-based grading of lumbar nerve root compromise due to disk herniation: reliability study with surgical correlation. Radiology 230: 583–588.

2. Ross JS (2004) Degenerative Disc Disease: Nomenclature. In: Ross JS, Brant-Zawadzki M, Moore K, Crim J, Chen M, editors. Diagnostic Imaging: Spine. 1 ed. Salt Lake City: Elsevier Health Sciences. pp. II-2-2–II-2-5.

3. Modic MT, Steinberg PM, Ross JS, Masaryk TJ, Carter JR (1988) Degenerative disk disease: assessment of changes in vertebral body marrow with MR imaging. Radiology 166: 193–199.

4. Pfirrmann CW, Metzdorf A, Zanetti M, Hodler J, Boos N (2001) Magnetic resonance classification of lumbar intervertebral disc degeneration. Spine 26: 1873–1878.

5. Weishaupt D, Zanetti M, Boos N, Hodler J (1999) MR imaging and CT in osteoarthritis of the lumbar facet joints. Skeletal Radiol 28: 215–219.
