# Supplementary material for: A Novel Tool for the Assessment of Pain: Validation in Low Back Pain
Source: PLoS Med. 2009 Apr 7;6(4):e1000047. doi: 10.1371/journal.pmed.1000047 (PMC2661253; doi:10.1371/journal.pmed.1000047)
Supplement: Text S1 — Standardized evaluation of pain (StEP). (0.10 MB PDF) [file pmed.1000047.s005.pdf]

Standardized Evaluation of Pain (StEP) **Neuropathic Pain**

Patient's name: .... Patient ID ....

Date of birth: ..... / ..... / ....

Examiner's name: ....

Date of the examination: ..... / ..... / ....

Introduction (please read to the patient)

To get a better understanding of what causes your pain, we would like to ask you a few questions. For example, we would like to know where your pain is located and how long it lasts. Please answer these questions by describing the pain that you have felt **during the last 24 hours**.

We will also perform a small number of tests. For example, we will test your response to touch or warm and cold temperatures. If these tests produce a painful sensation, we will ask you to describe the intensity of this pain using a scale from 0 (zero) to 10 (ten). An intensity of 0 means no pain; an intensity of 10 means pain as bad as you can imagine. Please rate your pain by indicating the number that best describes your pain.

Copyright © 2008 The General Hospital Corporation

INTERVIEW

1. **Location**

1.1	Is your pain superficial, e.g. located in the skin?	Yes ☐	No ☐
1.2	Is your pain deep, e.g. in muscles, bones or internal organs?	Yes ☐	No ☐

2. **Temporal characteristics**

2.1	Does your pain come and go in episodes that last for minutes or hours, with a pain-free period between these episodes?	Yes ☐	No ☐
2.2	What is the intensity of your pain during these episodes?	NRS ☐ Enter 0 (zero) if the pain does not manifest in episodes.	
2.3	Do you feel pain all the time?	Yes ☐	No ☐
2.4	What is the intensity of this ongoing pain?	NRS ☐ Enter 0 (zero) if there is no ongoing pain.	

3. **Quality**

Which of the following words would you choose to describe your pain?

3.1	☐ Burning	3.6	☐ Stabbing	3.11	☐ Cold
3.2	☐ Cramping	3.7	☐ Shooting	3.12	☐ Stinging
3.3	☐ Throbbing	3.8	☐ Squeezing	3.13	☐ Like an electric shock
3.4	☐ Sharp	3.9	☐ Dull	3.14	☐ Tender
3.5	☐ Pulling	3.10	☐ Painful pins and needles	3.15	☐ Spreading

4. **Pain evoked by activity or body position**

4.1	Is your pain caused by activity, e.g. when you are moving an arm or a leg, turning or bending your back, when you are walking, coughing or chewing?	Yes ☐	No ☐
4.2	Is your pain caused by a particular position of your body, e.g. when you are sitting or lying flat?	Yes ☐	No ☐

5. **Nonpainful sensations**

Do you suffer from unpleasant **nonpainful** sensations that are …

5.1	Tingling, like pins and needles?	Yes ☐	No ☐
5.2	Itching?	Yes ☐	No ☐
5.3	Warm or cold?	Yes ☐	No ☐

6. **Current pain**

6.1	Are you in pain right now?	Yes ☐	No ☐
6.2	What is the intensity of your pain now?	NRS ☐ Enter 0 (zero) if the patient is free of pain.	

PHYSICAL EXAMINATION

7. **Skin**

7.1	Swelling	Yes ☐	No ☐
	Skin color change:		
7.2	Reddening	Yes ☐	No ☐
7.3	Bluish skin color	Yes ☐	No ☐
7.4	Abnormal paleness	Yes ☐	No ☐
7.5	Abnormally dry skin	Yes ☐	No ☐
7.6	Excessive sweating	Yes ☐	No ☐
7.7	Trophic changes of the skin, hair or nails; or muscular atrophy that is not explained by denervation	Yes ☐	No ☐

Copyright © 2008 The General Hospital Corporation

8.	**Touch**		
8.1	Decreased response to stimulation with the low-strength von Frey filament	Yes ☐	No ☐
8.2	Pain evoked by stimulation with the low-strength von Frey filament	Yes ☐	No ☐
8.3	What is the intensity of pain evoked by the stimulation with the low-strength von Frey filament?	NRS ☐<br>Here and in the following tests enter 0 (zero) if pain is not evoked.	
8.1	Decreased response to stimulation with the high-strength von Frey filament	Yes ☐	No ☐
8.2	Pain evoked by stimulation with the high-strength von Frey filament	Yes ☐	No ☐
8.3	What is the intensity of pain evoked by the stimulation with the high-strength von Frey filament?	NRS ☐	
9.	**Blunt pressure**		
9.1	Decreased response to blunt pressure	Yes ☐	No ☐
9.2	Pressure-evoked pain	Yes ☐	No ☐
9.3	What is the intensity of the pressure-evoked pain?	NRS ☐	
10.	**Brush movement**		
10.1	Decreased response to brush movement	Yes ☐	No ☐
10.2	Brush movement-evoked pain	Yes ☐	No ☐
10.3	What is the intensity of the brush movement-evoked pain?	NRS ☐	
11.	**Vibration**		
11.1	Decreased response to vibration	Yes ☐	No ☐
12.	**Pinprick**		
12.1	Decreased response to pinprick	Yes ☐	No ☐
12.2	Excess pinprick-evoked pain	Yes ☐	No ☐
12.3	What is the intensity of the pinprick-evoked pain?	NRS ☐	
13.	**Warm temperature**		
13.1	Decreased response to warm temperature	Yes ☐	No ☐
13.2	Warm-evoked pain	Yes ☐	No ☐
13.3	What is the intensity of the warm-evoked pain?	NRS ☐	
14.	**Cold temperature**		
14.1	Decreased response to cold temperature	Yes ☐	No ☐
14.2	Cold-evoked pain	Yes ☐	No ☐
14.3	What is the intensity of the cold-evoked pain?	NRS ☐	
15.	**Temporal summation**		
15.1	An initially nonpainful response turns into pain during repeated stimulation	Yes ☐	No ☐
15.2	An initial painful response increases in intensity during repeated stimulation	Yes ☐	No ☐
16.	**Straight-leg-raising test**		
	Should only be performed in patients with back pain or pain in the lower extremities.		
16.1	Radicular pain produced by the straight-leg raising test	Yes ☐	No ☐

Copyright © 2008 The General Hospital Corporation

Standardized Evaluation of Pain (StEP) **Neuropathic Pain**
Instructions to examiners

General instructions

Use the standardized questions for the interview and follow the instructions given below for the physical examination. Examine the patient in a relaxed supine position on a firm, level couch. Perform each physical test in the painful area but demonstrate the procedure first in a neighboring or contralateral reference area free of pain.

Equipment

Two von Frey filaments, strength 2 g and 26 g

Pencil with a smooth-edged, nonmetal end

Make-up powder brush, width 1 cm

Standard tuning fork (128 Hz)

Safety pin

Two brass bars, diameter 10-15 mm

These brass bars will be needed for cold and warm stimulation. Keep the bars in two thermos vacuum flasks filled with water of **20°C** (68°F) and **40°C** (104°F) temperature, respectively. Check the water temperature before each test. Wipe the bars before use.

Alternatively, you can use a cylindrical Peltier thermode with a circular flat tip (diameter 10-15 mm) and set the temperature of the thermode to 20°C (68°F) for cold and 40°C (104°F) for warm stimulation.

Introduction

Read the introduction to the patient to explain the purpose and the structure of the pain assessment.

Interview and physical examination

INTERVIEW

1. **Location**

   If the patient describes a pain that is both superficial and deep, mark “Yes” for each location.

2. **Temporal characteristics**

   The essential distinction here is between intermittent pain episodes that are separated by intervals free of pain as opposed to ongoing pain during the last 24 hours. The duration of pain attacks may vary, e.g. from 1 or 2 minutes, as in *tics douloureux* in a patient with trigeminal neuralgia, up to several hours.

3. **Quality**

   Descriptions of more than one pain quality are allowed.

4. **Pain evoked by activity or body position**

   Distinguish between pain that is …

4.1 provoked by active movement.
4.2 elicited by passive maintenance of a particular body position.

5. **Nonpainful sensations**

   It is important to differentiate unpleasant nonpainful sensations (dysesthesiae) from pain, although patients may describe these sensations by using similar words, e.g. pins and needles.

6. **Current pain**

   This is an assessment of the patient’s current pain prior to the physical examination.

PHYSICAL EXAMINATION

7. **Skin**

7.7 Trophic changes include abnormally thin or thick skin, hair loss or abnormal hair growth, thick or brittle nails, and muscular atrophy that is not explained by denervation.

Copyright © 2008 The General Hospital Corporation

8. **Touch**

Use two von Frey filaments, of 2 g and 26 g strength, and apply each filament 4 times.

8.1 Consider the sense of touch intact (no decrease), when 3 out of 4 stimulations with a filament produce a normal response.

8.2 Record touch-evoked pain, when 3 out of 4 stimulations with a filament provoke pain.

9. **Blunt pressure**

Press the blunt eraser end of a pencil (diameter 5-8 mm) onto the skin so as just to indent the skin. Hold the pencil in this position for 10 seconds. The end of the pencil must not be made of metal to avoid the sensation of cold.

10. **Brush movement**

Move a make-up powder brush (width 1 cm) 3 times lightly over the skin, at 3-5 cm per second in a constant direction.

10.1 Record a decreased response to brush movement, when it is reproduced in 2 of the 3 movements.

10.2 Record brush movement-evoked pain, when pain is elicited by 2 out of the 3 movements.

11. **Vibration**

Place a standard tuning fork (128 Hz) **with** and **without** vibration on a prominent bone in the body area affected by the pain.

11.1 Response to vibration is decreased when the patient cannot differentiate between a vibrating and a nonvibrating tuning fork.

12. **Pinprick**

Use a medium-size safety pin and indent the skin with enough pressure to elicit a painful response on normal skin without leaving a mark.

12.1 The response to pinprick is decreased, when the patient describes only touch without a pricking sensation or no sensation at all in 3 out of 4 stimulations.

12.2 Record the intensity of the pain if it exceeds the intensity of the normal painful response to pinprick in the reference area in 3 out of 4 stimulations.

13. **Warm temperature**

Apply a warm stimulus by holding a brass bar or a thermode (diameter 10-15 mm) of 40°C (104°F) temperature onto the skin for 10 seconds. Do not apply pressure.

14. **Cold temperature**

Hold a brass bar or a thermode (diameter 10-15 mm) of 20°C temperature onto the skin for 10 seconds. Do not use pressure.

15. **Temporal summation**

Apply the stronger von Frey filament (26 g) repetitively on the skin at a rate of 1-2 times per second for 30 seconds.

Two positive outcomes are possible:

15.1 Although the first stimulus does not produce pain, pain occurs during repeated stimulation.

15.2 A painful response evoked by the first stimulus **increases in intensity** during repeated stimulation. Record a negative test result if the pain intensity does not increase beyond the intensity of the initial painful response.

16. **Straight-leg-raising test**

This test should only be performed in patients with back pain or pain in the lower extremities.

16.1 Raise the affected leg extended at the knee to a 90° angle unless elevation is limited by pain. Repeat the test, this time elevating the affected leg flexed at the knee. Reproduce the symptoms by raising the affected leg once again extended at the knee.

Consider only pain in a radicular distribution as a positive result, i.e. pain projecting into a leg dermatome (above or below the knee) when the leg is elevated extended at the knee. Pain caused by a radiculopathy usually decreases or is absent when the affected leg is raised with the knee flexed.

Copyright © 2008 The General Hospital Corporation
