# Supplementary material for: A Novel Tool for the Assessment of Pain: Validation in Low Back Pain
Source: PLoS Med. 2009 Apr 7;6(4):e1000047. doi: 10.1371/journal.pmed.1000047 (PMC2661253; doi:10.1371/journal.pmed.1000047)
Supplement: Text S2 — StEP score sheet for the distinction between radicular and axial low back pain. (0.07 MB PDF) [file pmed.1000047.s006.pdf]

Standardized Evaluation of Pain (StEP) **Neuropathic Pain**

Score sheet for the distinction between neuropathic and non-neuropathic low back pain

Patient's name: .... Patient ID ....

Date of birth: ..... / ..... / ....

Examiner's name: ....

Date of the examination: ..... / ..... / ....

Radicular versus axial low back pain

StEP may be used to help discriminate between neuropathic (radicular) and non-neuropathic (axial) low back pain. The score sheet below lists interview questions and physical tests included in StEP that assess symptoms and signs of radicular back pain. Transfer responses to these questions and test results from the previous pages of StEP and add the scores.

If the total score is ≥ 4, low back pain is likely to be radicular.
A total score < 4 indicates axial low back pain.

				Score
	INTERVIEW			
2.3	**Pain all the time?**	Yes	☐	-2
		No	☐	0
3.	**Pain quality**			
	Burning (3.1) or cold (3.11), or both	Yes	☐	-1
		No	☐	0
5.	**Nonpainful sensations** (any)	Yes	☐	1
		No	☐	0
	PHYSICAL EXAMINATION			
7.	**Skin changes** (any)	Yes	☐	-3
		No	☐	0
9.	**Blunt pressure**			
	Decreased response to blunt pressure or pressure-evoked pain	Yes	☐	1
		No	☐	0
10.	**Brush movement**			
	Decreased response to brush movement or brush movement-evoked pain	Yes	☐	-2
		No	☐	0
11.1	**Decreased response to vibration**	Yes	☐	1
		No	☐	0
12.	**Pinprick**			
	Decreased response to pinprick or excess pinprick-evoked pain	Yes	☐	2
		No	☐	0
14.	**Cold temperature**			
	Decreased response to cold temperature or cold-evoked pain	Yes	☐	3
		No	☐	0
15.	**Temporal summation**			
	A nonpainful response turns into pain or pain intensity increases during repeated stimulation	Yes	☐	-1
		No	☐	0
16.1	**Radicular pain produced in the straight-leg-raising test**	Yes	☐	7
		No	☐	0
		**Total score**		

Copyright © 2008 The General Hospital Corporation
